# Treatment of tardive dyskinesias with vitamin E: A case series

**DOI:** 10.1192/j.eurpsy.2023.2132

**Published:** 2023-07-19

**Authors:** B. Senol, F. N. Akarca, R. N. Ekinci, E. Goka

**Affiliations:** Psychiatry, Ankara City Hospital, Ankara, Türkiye

## Abstract

**Introduction:**

Tardive dyskinesia is usually persistent, irreversible involuntary movement of the tongue, lips, face, trunk and extremities in patients taking long-term dopaminergic antagonist drugs. Although it is mostly associated with the use of neuroleptics, cases of tardive dyskinesia existed before the discovery of these agents. Patients with schizophrenia and other neuropsychiatric disorders are particularly at risk for tardive dyskinesia because they are exposed to neuroleptics and anticholinergic agents for longer periods than healthy individuals. Free radicals are thought to be probably involved in the pathogenesis of tardive dyskinesia. Vitamin E is a fat-soluble antioxidant, and it is thought to be effective in the treatment of antipsychotic-associated tardive dyskinesia, as it has a cytotoxic free radical-binding effect.

**Objectives:**

In this poster presentation, it was aimed to evaluate the clinical results of the treatment of tardive dyskinesia with high-dose vitamin E in four inpatients with serious mental illness and long-term antipsychotic use. In addition, the treatment of tardive dyskinesia will be discussed in the light of current literature data.

**Methods:**

In the case report, there are three patients with schizophrenia and one with mild mental retardation. They were treated with 1600 IU of vitamin E per day. The patients continued their vitamin E treatment for 90 days. The severity of tardive dyskinesia of the patients was measured by Abnormal Involuntary Movement Scale (AIMS).

**Results:**

At the end of the 90 day treatment, the AIMS measurements of the subjects decreased 72,7%, 73,3%, 72,2% and 80% respectively.

**Image:**

**Figure:**
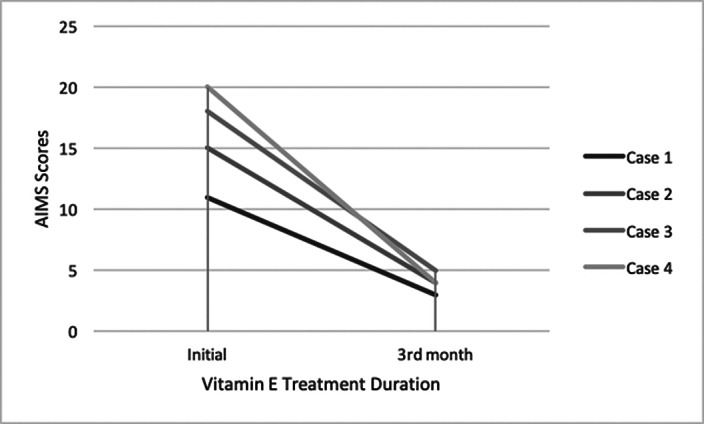

**Conclusions:**

In our clinic, we observed that patients using long-term typical antipsychotics were prescribed antipsychotics in unindicated situations or in high doses, patients with high risk for tardive dyskinesia were not taken into consideration when planning treatment, and we encountered cases of tardive dyskinesia despite the widespread use of atypical antipsychotic drugs in hospitalized patients. The use of benzodiazepines is restricted especially in elderly individuals due to their side effects and the risk of addiction in long-term use. Although the clinical importance of vitamin E is unknown, it is preferred because it can be used with a low risk of side effects, considering that it can prolong bleeding time. Although the results of a review of tardive dyskinesia treatment do not suggest that vitamin E reliably improves tardive dyskinesia symptoms, our experience shows that patients benefit from vitamin E treatment. In this regard, there is a need for studies that will be conducted with a large sample and compare the effectiveness of vitamin E with the treatments known to be effective in tardive dyskinesia.

**Disclosure of Interest:**

None Declared

